# Bioluminescence-Based Determination of Cytosolic Accumulation of Antibiotics in *Escherichia coli*

**DOI:** 10.1021/acsinfecdis.3c00684

**Published:** 2024-04-09

**Authors:** Rachita Dash, Kadie A. Holsinger, Mahendra D. Chordia, Mohammad Sharifian Gh., Marcos M. Pires

**Affiliations:** Department of Chemistry, University of Virginia, Charlottesville, Virginia 22904, United States

**Keywords:** antibiotic, bacterial, cellular, permeability, methods, drug molecules

## Abstract

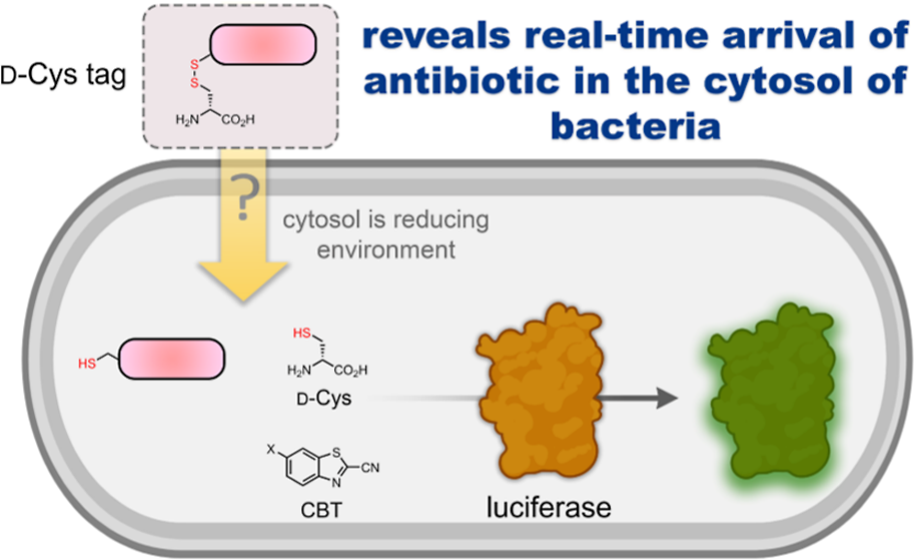

Antibiotic resistance is an alarming public health concern that affects millions of individuals across the globe each year. A major challenge in the development of effective antibiotics lies in their limited ability to permeate cells, noting that numerous susceptible antibiotic targets reside within the bacterial cytosol. Consequently, improving the cellular permeability is often a key consideration during antibiotic development, underscoring the need for reliable methods to assess the permeability of molecules across cellular membranes. Currently, methods used to measure permeability often fail to discriminate between the arrival within the cytoplasm and the overall association of molecules with the cell. Additionally, these techniques typically possess throughput limitations. In this work, we describe a luciferase-based assay designed for assessing the permeability of molecules in the cytosolic compartment of Gram-negative bacteria. Our findings demonstrate a robust system that can elucidate the kinetics of intracellular antibiotic accumulation in live bacterial cells in real time.

Antibiotic resistance is a growing global problem that directly leads to increased risks associated with bacterial infections. Recent data reveal that antibiotic resistance was responsible for nearly 5 million fatalities in 2019.^1^ A primary driver of the resistance phenotype is the overuse and misuse of antibiotics in human medicine and agriculture, as well as the lack of development of new antibiotics.^2^ Infections that are highly resistant can lead to prolonged illness, increased healthcare costs, and higher mortality rates. Urgent measures, including responsible antibiotic stewardship, innovative drug development, and public awareness, are essential to combating this pressing threat to modern medicine. Consequently, it is important to prioritize strategies aimed at circumvention of antibiotic resistance.

Among the primary hurdles in the development of effective antibiotics is their general lack of cellular permeability.^3^ This challenge is particularly pronounced when targeting Gram-negative bacteria and mycobacteria due to their additional membranes that pose barriers to molecular entry. Compounding this issue is the fact that some of the most crucial drug targets are situated within the bacterial cytosol, emphasizing the need for permeable antibiotics in combatting bacterial infections.^4^ In the pursuit of novel antibiotics, it becomes critically important to develop methodologies that can reliably report on molecule accumulation in bacteria with high efficiency.^5−12^ Currently, several methods are available to evaluate the permeability of molecules in Gram-negative bacteria. The most widely used method of LC–MS/MS does not require a chemical tag to be added to the test molecule.^13,14^ However, its widespread adoption has been significantly hampered by inherent throughput limitations, limiting its broad application in the field. Another widely used approach involves optical analysis of cells that are treated with compounds chemically modified with a fluorophore to track their entry into the cell.^15,16^ Critically, these methods often fail to report on whether the molecules arrive within the cytoplasmic space and, instead, provide information on the total association of the molecules with the target cells.^17^

Our group has recently described methods to interrogate the accumulation of molecules onto the surface of Gram-positive bacteria and past the outer membrane of diderm bacteria using a combination of click chemistry^18,19^ and HaloTag.^20^ Herein, we sought to establish a luciferase-based assay^21^ to determine the accumulation of molecules to the cytosol of Gram-negative bacteria in real time. In this assay, luciferase-expressing bacteria in the presence of 6-hydroxy-2-cyanobenzothiazole (CBT–OH) are incubated with test molecules tagged via a disulfide bond to d-cysteine (d-cys)^22^ (Figure 1a). Upon the arrival of the conjugate in the reducing environment of the cytosol,^23^ reduction of the disulfide bond to generate d-cys in the cytosol enables a fast and biorthogonal^24^ recombination of CBT and d-cys to generate intracellular d-luciferin that is rapidly processed by luciferase to generate light (Figures 1a; S1).^25^ We showed that the system was robust, displayed a high signal-to-noise ratio, and revealed the kinetics of the intracellular accumulation of antibiotics.

**Figure 1:**
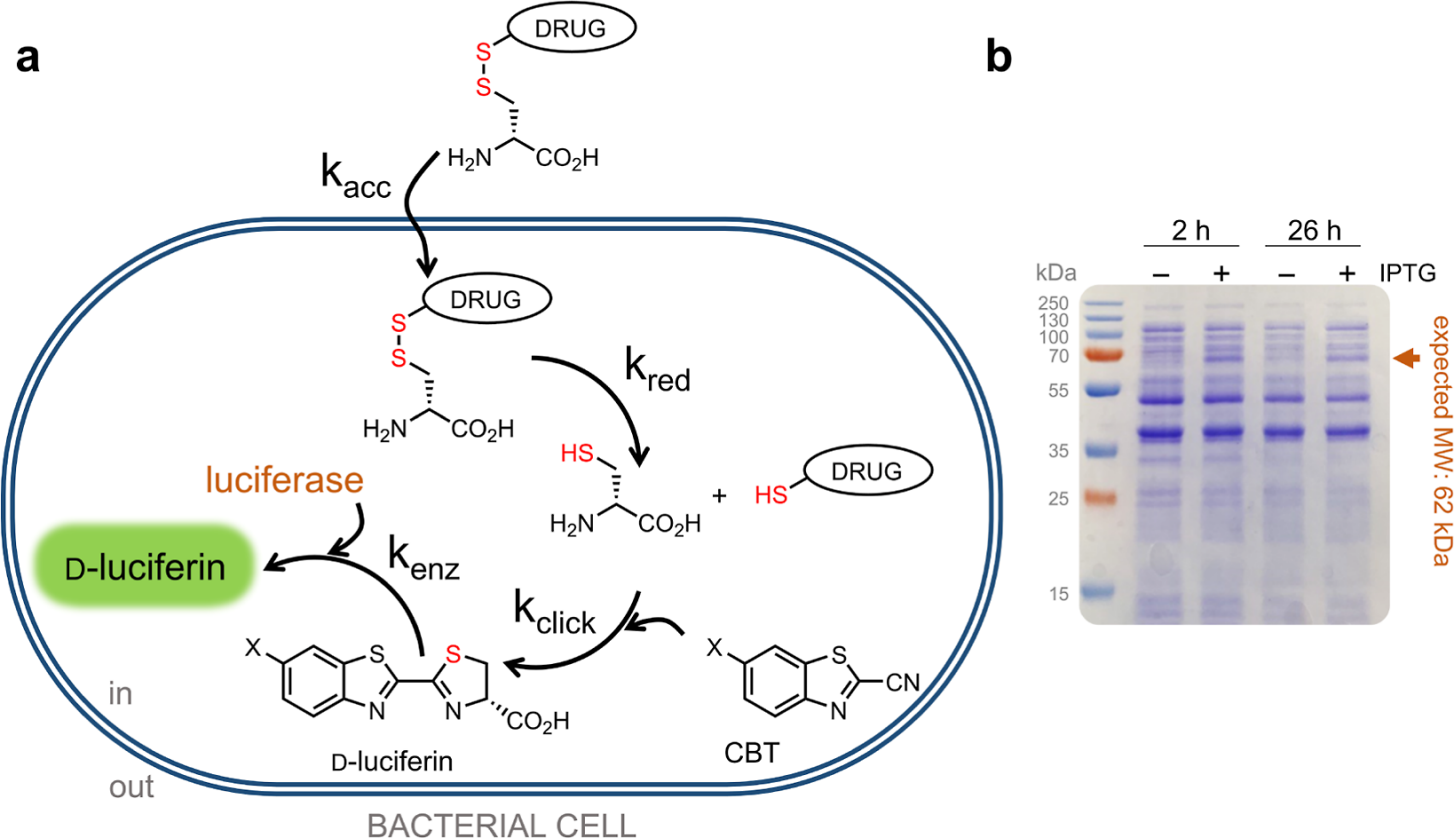
(a) Schematic showing the workflow of DCCAA. (b) SDS-PAGE analysis of luciferase protein expression in *E. coli* at 2 or 26 h post IPTG induction. A molecular weight ladder with sizes in kilodaltons (kDa) is shown. The expected molecular weight of luciferase is 62 kDa.

## Results and Discussion

The reaction between aminothiols (including cysteines) and CBT exhibits numerous favorable characteristics, including reaction speed, ease of use, and exceptional specificity. This reaction has been leveraged in a variety of applications including protein labeling,^26−28^ molecular imaging,^29^ and to investigate the permeability of peptides into mammalian cells.^21^ Alternatively, mice expressing luciferase have been used to detect endogenous d-cys in the brain of animals.^30,31^ We identified many advantages of the d-cys cytosolic accumulation assay (DCCAA). DCCAA generates real time measurement upon singular cellular treatment (no washing steps or chase treatment required), thereby reducing the number of assay manipulation steps. Additionally, the assay workflow is compatible with multiwell plates thus enabling high-throughput analysis. Finally, bioluminescence signals are more compatible with bacterial species that have intrinsic fluorescence, which can introduce a high background noise in fluorescence-based assays.

The first step was to evaluate the expression of luciferase in *Escherichia coli*. Bacterial cells transformed with the luciferase-expressing plasmid were grown to mid log phase and induced with isopropyl-β-D-1-thiogalactopyranoside (IPTG). Two conditions were tested: a 2 h short induction and a 26 h long induction.^32,33^ Both induced and uninduced samples were collected, and protein expression was evaluated via sodium dodecyl sulfate polyacrylamide gel electrophoresis (SDS-PAGE). The presence of a band consistent with the molecular weight of luciferase (62 kDa) was identified (Figure 1b). Our results showed similar expression levels for both time points; therefore, the shorter incubation time (2 h) was selected for subsequent assays.

**Figure 2:**
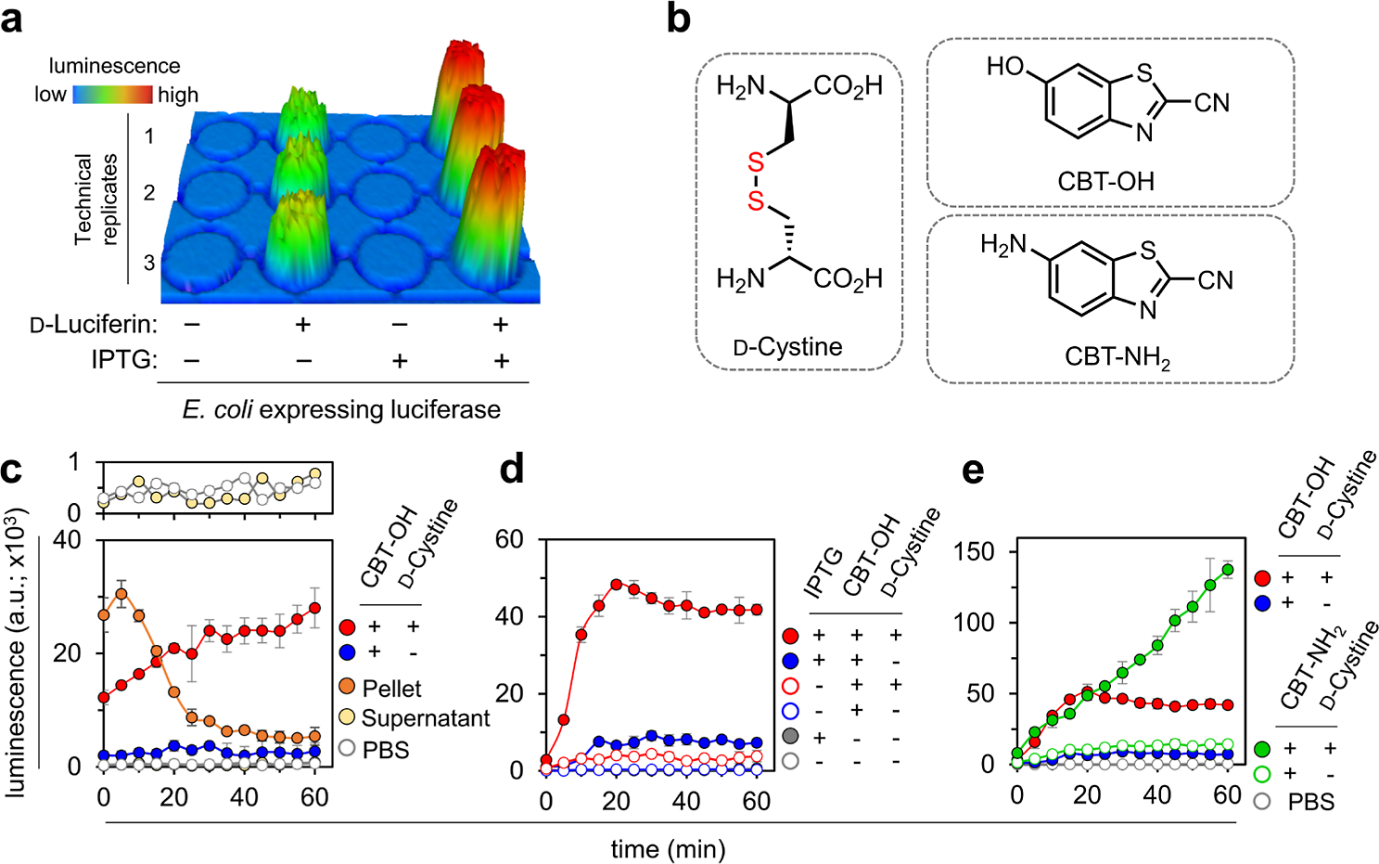
(a) Bioluminescence image of a 96-well plate containing *E. coli* treated with100 μM d-luciferin in the presence and absence of IPTG induction. (b) Chemical structures of the building blocks used for the assay. (c) Bioluminescence analysis of luciferase-expressing *E. coli* cells incubated with 100 μM CBT–OH and 100 μM d-cystine for 30 min, followed by centrifugation and separation of supernatant and pellet at which point luminescence measurements were initiated and continued for 60 min. Noncentrifuged cells (CBT–OH + d-cystine) were used as a control. Inset at the top displays a magnified portion of the graph, highlighting the PBS and supernatant traces. (d) Luciferase-expressing *E. coli* cells treated with 100 μM CBT–OH and 100 μM d-cystine in the presence and absence of IPTG induction, over 60 min. (e) Luciferase-expressing *E. coli* cells treated with 100 μM CBT–OH or 100 μM CBT–NH_2_ and 100 μM d-cystine over 60 min. Cells treated with PBS and CBT only (CBT–OH or CBT–NH_2_) were used as controls wherever appropriate. Data are represented as mean ± SD (*n* = 3 independent samples in a single experiment).

An assessment of the difference in luminescence signal between induced and uninduced samples in DCCAA was then performed. Bioluminescence imaging of a multiwell plate containing luciferase-expressing *E. coli* cells treated with d-luciferin also revealed a notable increase in luminescence upon induction (Figure 2a). *E. coli* cells harboring the luciferase plasmid were coincubated with CBT–OH and d-cystine for one h, during which luminescence was recorded. Here, d-cystine served as a surrogate for a test molecule (Figure 2b). Kinetic analysis of the cellular treatment revealed a pronounced increase in the luminescence signal over time. The signal response was IPTG-dependent, which is consistent with the proposed luciferase mediated processing of d-luciferin upon generation of d-cys in the cytosol.

Next, we set out to establish whether the luminescence signal generation was confined to within the cellular structure instead of the extracellular recombination of CBT/d-Cys. Luciferase-expressing*E. coli* cells were first coincubated with CBT–OH and d-cystine for 30 min, after which, the cells were subjected to centrifugation, leading to the separation of the supernatant from the cellular pellet. The supernatant was then resuspended in phosphate-buffered saline (PBS). Luminescence measurements were then performed for an additional 60 min (Figure 2c). Consistent with the expected cytosolic localization of the luciferase, the luminescence signal detected in the supernatant was minimal, whereas the signal originating from the cellular pellet was significantly more pronounced. These observations are consistent with the intracellular nature of the signal and provide evidence that the luciferase enzyme remains localized within the cytoplasm for the entire duration of the assay.

We appreciated that the physicochemical properties of CBT could potentially be subjected to its own cellular accumulation barriers. We therefore sought to test two versions of CBT that have been described as compatible with recombination with d-cys to form luciferin. We evaluated CBT–OH and 6-amino-2-cyanobenzothiazole (CBT–NH_2_)^34^ as potential substrates for the click reaction (Figure 2b,e). Notably, the in vitro rate constants for the reactions of the hydroxy- and amino-cyanobenzothiazole with l-cys have previously been reported to be 3.2 and 2.6 M^–1^ s^–1^, respectively.^35^ Briefly, luciferase-expressing *E. coli* cells were coincubated with the CBT variants and d-cystine as described before. Our results showed that cells treated with CBT–NH_2_ produced a higher luminescence signal than those treated with CBT–OH (Figure 2d). Interestingly, CBT–OH outperformed CBT–NH_2_ in an in vitro cell-free setup consistent with prior reports (Figure S2). We pose that CBT–NH_2_ may have higher levels of accumulation relative to its hydroxy counterpart, and it was therefore selected as the preferred CBT variant for subsequent experiments.

**Figure 3:**
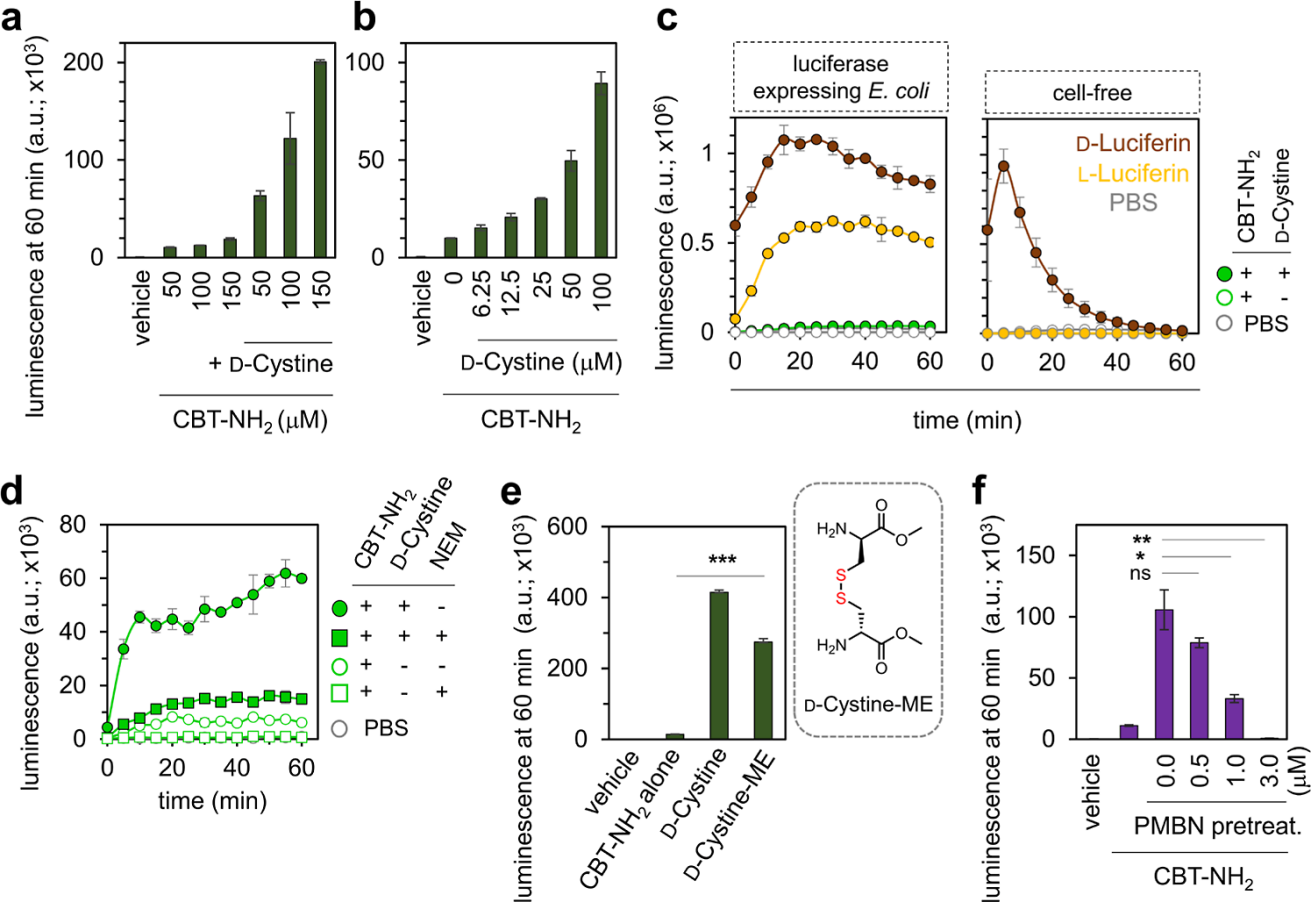
(a) Bioluminescence analysis of luciferase-expressing *E. coli* cells treated with 50 μM d-cystine and varying concentrations of CBT–NH_2_ over 60 min at the 60 min time point. (b) Luciferase-expressing *E. coli* cells treated with 100 μM NH_2_–CBT and varying concentrations of d-cystine at the 60 min time point. (c) Luciferase-expressing *E. coli* cells (left) or a cell-free assay with luciferase enzyme (right) treated with 10 μM l-luciferin, 10 μM d-luciferin or 10 μM d-cystine with 100 μM CBT–NH_2_ (cell assay), over 60 min. (d) Luciferase-expressing *E. coli* cells treated with 100 μM d-cystine and 100 μM CBT–NH_2_ or 100 μM CBT–NH_2_ only, in the presence and absence of a 30 min pretreatment with 100 μM NEM, over 60 min. (e) Luciferase-expressing *E. coli* cells treated with 100 μM d-cystine or 100 μM d-cystine-ME and 100 μM CBT–NH_2_ at the 60 min time point. (f) Luciferase-expressing *E. coli* cells treated with 100 μM d-cystine and 100 μM CBT–NH_2_ in the absence and presence of a 30 min pretreatment with PMBN at different concentrations at the 60 min time point. Cells treated with only PBS and CBT–NH_2_ were used as controls, wherever appropriate. Data are represented as mean ± SD (*n* = 3 independent samples in a single experiment). Statistical analysis performed by two-tailed *t*-test with Welch’s correction, *p ≤ 0.01, **p ≤ 0.01, ***p ≤ 0.001, ns = not significant.

We then set out to empirically determine the optimum concentration of CBT–NH_2_ for DCCAA. Briefly, following IPTG induction, bacterial cells were washed and either coincubated with varying concentrations of CBT–NH_2_ and 50 μM d-cystine or incubated with CBT–NH_2_ alone. A concentration of 100 μM was considered to have a sufficient signal-to-noise ratio necessary for the assay and was selected for subsequent experiments (Figure 3a). This concentration of CBT–NH_2_ was also found to not alter the cellular viability as determined by colony forming units (CFUs) analysis (Figure S3). Next, a similar titration experiment was performed with d-cystine by using a constant level of CBT–NH_2_. Our results showed that both 50 and 100 μM d-cystine exhibited a favorable signal-to-noise ratio (Figure 3b).

Through our initial assay development efforts, the background signal was higher than we had anticipated; therefore, we sought to investigate its potential source. We considered that it could be from intracellular pools of l-cys combining with CBT–NH_2_ to form l-luciferin (Figure S4).^21,29,36−38^ Previous reports have shown that l-luciferin can undergo epimerization in the presence of firefly luciferase and then act as a substrate for the enzyme.^39^ Indeed, upon coincubation of luciferase-expressing *E. coli* with L-luciferin, a signal lower than that of d-luciferin, yet significantly higher than the background, was observed (Figure 3c). This observation suggests that l-luciferin undergoes epimerization within the cytoplasm of *E. coli*. In a cell free experiment, l-luciferin exhibited a baseline luminescence signal in the presence of purified luciferase, whereas d-luciferin exhibited a considerably elevated signal intensity (Figure 3c). Notably, this observation is in agreement with the essential role of Coenzyme A (CoA) in the epimerization process of l-luciferin to d-luciferin catalyzed by luciferase (Figure S5).^37,40^

We next sought to evaluate the necessity for d-cys to be uncoupled prior to signal generation. At first, a cell free set up was evaluated by incubating d-cystine with luciferase in the presence or absence of the reducing agent tris(2-carboxyethyl) phosphine (TCEP) (Figure S6). The inclusion of TCEP was found to be essential for signal generation, providing evidence for the requirement of a reducing environment to promote signal production. A similar requirement for a reducing environment was next evaluated in the cell. For these experiments, *E. coli* was treated with N-ethylmaleimide (NEM), which covalently modifies cellular thiols and is expected to reduce the cellular pool of reducing agents including glutathione and l-cys.^23,41^ Luciferase-expressing cells were pretreated with NEM or PBS, followed by incubation with d-cystine, as described previously. Pretreatment with NEM resulted in a complete shutdown of the signal (Figure 3d). These results demonstrate that the reducing environment of the cell is required for signal generation in a manner that is consistent with the levels of cellular thiols. Moreover, we tested the effect of NEM on the background signal that emerged solely from the addition of CBT–NH_2_. Our findings demonstrate that NEM also effectively abolished the signal originating from CBT–NH_2_ (Figure 3d), indicating that the presence of l-cys most likely contributes to the observed background signal.

Considering the inherent characteristics of our initial test compound, d-cystine, containing two carboxylic acid moieties, we explored the possibility of masking these groups to enhance the accumulation. Masking negatively charged carboxylic acids through esterification is widely used as a permeability strategy to enhance the lipophilicity and passive membrane permeability of molecules, particularly, therapeutic agents with intracellular targets.^42,43^ Once inside the cell, the ester may be enzymatically hydrolyzed to the acid, resulting in conversion to the parent compound. It is noteworthy that the introduction of a methyl group at the carboxylic position in d-luciferin, as seen in d-luciferin-methyl-ester, results in its failure to be recognized by the firefly luciferase enzyme.^44^ While the findings of Antonczak and colleagues^45^ suggest an absence of methyl esterases in *E. coli*, subsequent research by the Grimes laboratory^46^ utilizing methyl ester NAM derivatives in their peptidoglycan labeling approach, lends support to the presence of methyl esterases in *E. coli*.

To test the masking ability of methyl ester, we used DCCAA to compare d-cystine and d-cystine-methyl ester (d-cystine-ME) (Figure S7). Our results showed that cellular treatment with d-cystine-ME led to signals well above the background, suggestive of esterase unmasking of the methyl ester (Figure 3e). Interestingly, the cellular signals were ca. 30% lower than that of unmasked d-cystine. This could indicate that d-cystine may be actively transported by an importer^47,48^ or the esterase processing is inherently slow. When the experiment was conducted in a cell-free system, the signal from d-cystine-ME remained at the background level (Figure S8). These findings provide support for the processing of ester groups in *E. coli* cells. The presence of esterases in *E. coli* bears relevance in the realm of drug development, especially in the potential utilization of a prodrug approach for antibiotics. Nevertheless, we believe that DCCAA can be generally leveraged to gain further insight into the substrate specificity and enzymatic activity of *E. coli* esterases.

The diderm cell envelope structure in Gram-negative bacteria, particularly the outer membrane, serves as a substantial barrier to the permeation of antibiotics with intracellular targets. Therefore, there is a critical need to identify and develop molecules that can disrupt this accumulation barrier. Such molecules can potentially be used as antibiotic adjuvants that can broadly improve activity. One example of a molecule capable of perturbing the outer membrane is polymyxin B nonapeptide (PMBN), which is a modified form of Polymyxin B lacking the fatty acid tail. PMBN can permeabilize the outer membrane of *E. coli* at low, nontoxic concentrations.^49^ Moreover, it has also demonstrated the ability to enhance the efficacy of erythromycin and provide protection against Gram-negative bacteria in mice.^50^ We posed the question of whether the inclusion of PMBN in our assay would increase the permeability of bacterial cells to d-cystine, leading to an amplified signal response.

To test the potential impact of PMBN, luciferase expressing *E. coli* cells were pretreated with PMBN at increasing concentrations and the assay was carried out as described previously (Figure 3f). Unexpectedly, a dose-dependent decrease in the luminescence signal with increasing concentrations of PMBN was observed. This trend was also observed when cells were, instead, treated with d-luciferin (Figure S9). Crucially, we noted no loss of cellular viability with the same concentrations of PMBN (Figure S10). We wondered whether PMBN could be leading to the release of components critical to DCCAA. We observed a dose-dependent increase in outer membrane permeation as measured by nitrocefin (Figure S11). A similar pattern was observed in the case of a SYTOX Green assay, which is a high-affinity nucleic acid stain typically used to assess membrane integrity (Figure S12). Notably, treatment of *E. coli* cells with PMBN has been previously reported to cause the release of intracellular, low-molecular weight substances such as free amino acids and uracil.^51^ Hence, we hypothesize that the observed reduction in signal may be due to the leakage of substrates, specifically the release of ATP from the cells or the *in cyto* produced d-luciferin which is integral for the oxidation of d-luciferin by luciferase.^52,53^

**Figure 4:**
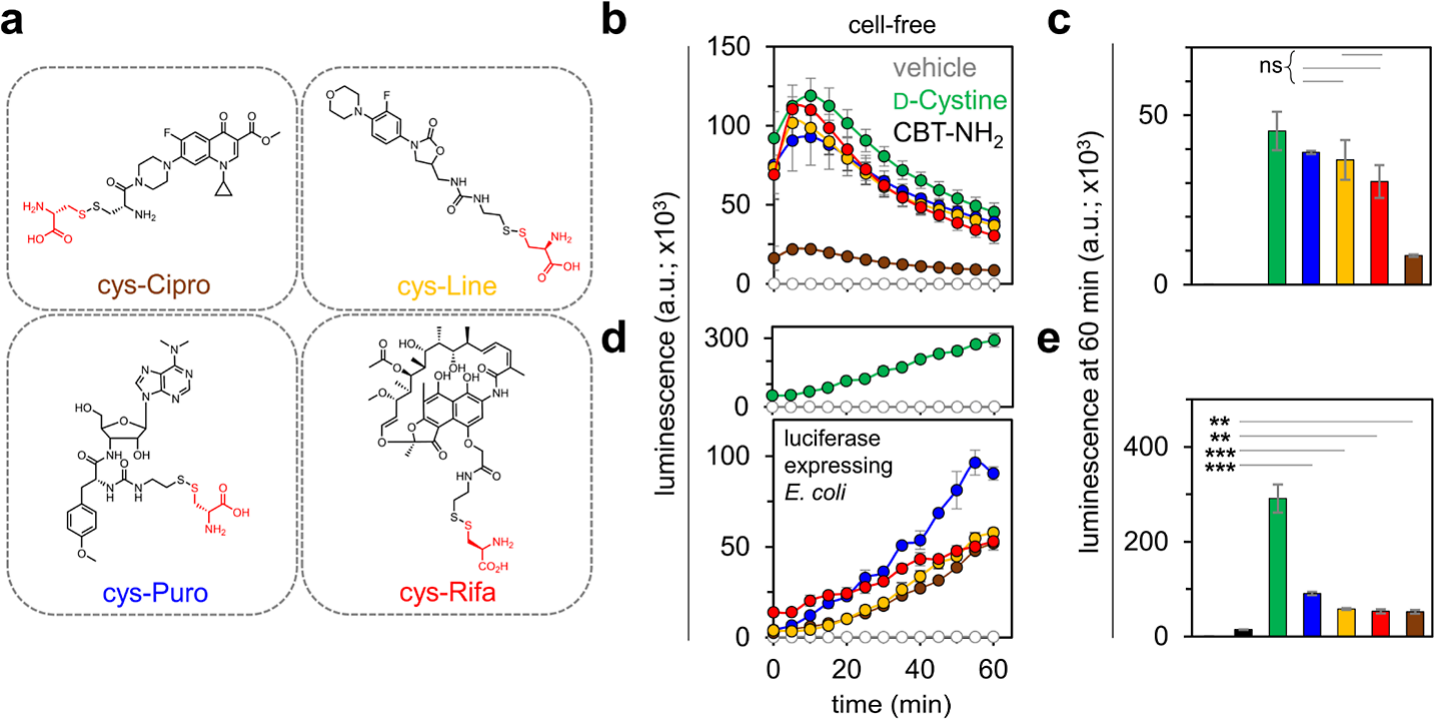
(a) Structures of the antibiotic conjugates tagged with a d-cys moiety via a disulfide linkage. (b) Bioluminescence analysis of a cell-free assay with luciferase enzyme treated with 25 μM d-cystine or 25 μM antibiotic conjugates and 25 μM CBT–NH_2_ over 60 min and (c) at the 60 min time point upon treatment with 1 mM TCEP. (d) Bioluminescence analysis of luciferase-expressing *E. coli* cells treated with 50 μM d-cystine or 50 μM antibiotic conjugates with 100 μM CBT–NH_2_ over 60 min and (e) at the 60 min time point. The inset at the top of (d) provides a zoomed-out view of the graph, highlighting the d-cystine signal. Cells treated with PBS and CBT only (CBT–NH_2_) were used as controls wherever appropriate. Data are represented as mean ± SD (*n* = 3 independent samples in a single experiment). Statistical analysis performed by two-tailed *t*-test with Welch’s correction, **p ≤ 0.01, ***p ≤ 0.001, ns = not significant.

Having optimized the assay parameters of DCCAA and shown its ability to respond to the model molecule d-cystine, we next leveraged this assay to evaluate the accumulation of structural motifs that are relevant for clinical applications. For this, a small panel of antibiotics (including ciprofloxacin, puromycin, linezolid, and rifamycin B) were synthesized and tagged with a disulfide-linked d-cys (Figure 4a). In all, this panel included a range of molecules that varied in physicochemical properties. Before performing the cellular assay, we aimed to test the release of d-cys in a cell-free experiment. It is noteworthy that in the absence of a permeability barrier, when exposed to a reducing agent, all four compounds were expected to generate an identical luminescence signal at the same concentration. This should be because of the release of an equal number of d-cys molecules from each compound.

Interestingly, our cell-free results revealed an unexpected result (Figure 4b). While there was no significant difference in the luminescence signal for puromycin, rifamycin B, and linezolid conjugates, the signal for ciprofloxacin was much lower (Figure 4c). We hypothesized that the residual covalently tagged d-cys in the ciprofloxacin conjugate after the breakage of the disulfide bond, could potentially undergo a nonproductive click reaction with CBT–NH_2_ (Figure S13) thereby reducing the effective concentration of CBT. Nonetheless, all four antibiotics were evaluated in the cellular assay. As before, the evaluation of these molecules was conducted by coincubating luciferase-expressing bacterial cells with CBT–NH_2_ and the antibiotic conjugates, and subsequent luminescence measurements were made. It is noteworthy that all four conjugates demonstrated noticeable permeation exceeding the baseline signal, as seen in Figure 4d, and as indicated by the luminescence signals observed at the 60 min time point (Figure 4e). A similar pattern was also observed when the observation window was extended to 120 min (Figure S14) and we demonstrate that the assay is concentration dependent (Figure S15). Notably, addition of the antibiotic conjugates to the luciferase expressing *E. coli* cells was found to be minimally disruptive to their cell envelope structure, as evidenced by a SYTOX Green assay (Figure S16).

We then proceeded to develop a kinetic model for the molecular uptake of the conjugated antibiotics. The model serves as the foundation for a nonlinear least-squares analysis of our data. Specifically, the model accounts for the accumulation of each molecule in the cytoplasm of *E. coli* and the subsequent click and luciferase-based bioluminescence enzymatic reactions. Of significance, the time-dependent luminescence response (I_Lum._) can be characterized by eq 1, where ε_0_ and I_0_ respectively represent the luminescence coefficient factor and baseline signal in our experiments. d-Luciferin (*) denotes the oxidized d-luciferin molecule that is associated with the luminescence signal.
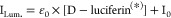
1

Time-dependent luminescence signals are then utilized to derive a solution with physical significance for the specified differential equations, as detailed in Supporting Information. Special attention is given to estimating the accumulation rate constant, denoted as *k*_acc_ (min^–1^), and the reduction rate constant, denoted as *k*_red_ (min^–1^). Values for the click reaction rate constant, *k*_click_ (156 M^–1^min^–1^)^35^ and the enzymatic reaction rate constant, *k*_enz_ (96 min^–1^)^54^ were adopted from the literature.

**Figure 5:**
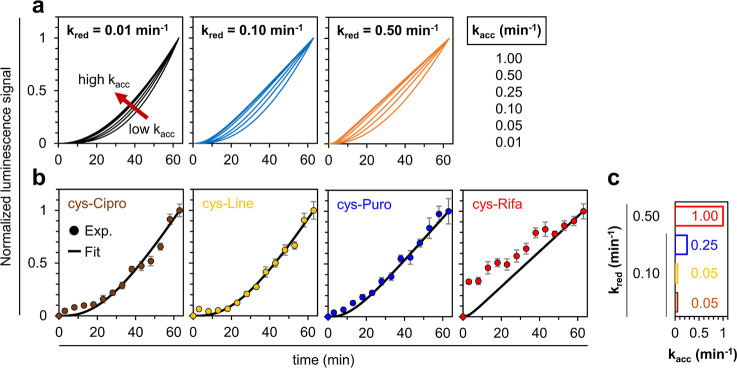
(a) Simulated luminescence traces, based upon the model for molecular accumulation in bacteria, with gradually increasing rate constants *k*_acc_ and *k*_red_. (b) Experimental time-resolved luminescence traces illustrating the accumulation of test molecules in *E. coli*. The best-fit lines from simulations are overlaid in black. The corresponding values for *k*_acc_ and *k*_red_ for each, are depicted in (c).

To illustrate how *k*_acc_ and *k*_red_ affect time-dependent luminescence signals, we selectively adjusted the associated transport rates in a set of simulated kinetic responses. In Figure 5a, a sequence of simulated responses is presented, incorporating various *k*_acc_ values ranging from 0.01 to 1.00 min^–1^, paired with each *k*_red_ value set at 0.01, 0.10, and 0.50 min^–1^. Importantly, an offset of 3 min was introduced in the experimental data to account for the time delay between the beginning of the assay and the first luminescence reading, owing to logistics of pipetting, mixing, etc. Additionally, all signals, experimental and simulated, were normalized with respect to their values at 63 min for the purpose of this model. As depicted, irrespective of the *k*_red_ values, the curves exhibit exponential behavior at lower *k*_acc_ values, transitioning gradually into a semilinear shape at higher *k*_acc_ values.

Next, we sought optimal fits for each time-resolved signal, representing the averages from four independent trials. In Figure 5b, the most accurate fitting curves are presented, superimposed on each signal. Notably, for the ciprofloxacin-methyl-ester, linezolid, and puromycin conjugates, the best-estimated *k*_acc_ values were respectively determined to be 0.05, 0.05, and 0.25 min^–1^, each accompanied by a *k*_red_ of 0.10 min^–1^ (Figure 5c). These findings imply that the membrane transport rates for conjugated ciprofloxacin methyl ester and linezolid are five times lower than that of conjugated puromycin. Significantly, despite linezolid being known for its vulnerability to efflux pump activity, we observed its accumulation behavior in our assay. This may be attributed to the possibility that our luminescence readout process operates on a faster time scale than its efflux out of the cell or that efflux pumps do not effectively recognize linezolid when tagged with a d-cys moiety. As far as d-cys is concerned, as expected, it displayed a higher *k*_acc_ value of 1.00 min^–1^ and *k*_red_ value of 0.50 min^–1^, suggesting faster accumulation and reduction processes, compared to the conjugated antibiotics (Figure S17). Unexpectedly, rifamycin B, when compared to the other three components, displayed slightly distinct kinetics. The best-fitted curve revealed a rate constant similar to that of d-cystine (i.e., *k*_acc_ = 1.00 min^–1^; *k*_red_ = 0.50 min^–1^). This may be due to a difference in its membrane transport mechanism or as a result of the property of the molecule itself given its larger size. Significantly, molecules with comparable molecular weights to our antibiotic conjugates, such as malachite green (329.46 Da), have been documented to exhibit a transport rate of approximately 4.2 min^–1^ and 0.013 min^–1^ through the outer- and inner-membranes of *E. coli*, respectively.^55^ In contrast, a similar molecule without a permanent dipole moment, namely crystal violet (372.54 Da; net charge of 1+), displays inner-membrane transport rates that can be orders of magnitude smaller.^56^

## Discussion and Conclusion

Quantifying intracellular compound accumulation within the bacterial cytosol, especially during the development phase of antibiotics with cytosolic targets, is crucial for the development of efficacious drugs with useable potency against their targets. Here, we present DCCAA, a luminescence-based method enabling the real-time monitoring of antibiotic intracellular accumulation in live Gram-negative bacterial cells. In this technique, the molecule of interest is conjugated with a d-cys moiety through a disulfide bond, which is susceptible to reduction within the cytosolic milieu. The liberated d-cys then engages in a click reaction with CBT, coadministered with the tagged molecule, resulting in the generation of d-luciferin. This substrate undergoes oxidation by luciferase, emitting light in the process, as a measurable indicator of intracellular antibiotic accumulation dynamics (Figure 1a). Additionally, our results showed compelling evidence supporting the presence of methyl esterases in *E. coli*. This investigation highlights the potential use of DCCAA to decipher the presence and substrate-specificity of esterases in different bacterial species. We were also able to elucidate the membrane disruption activity of an antimicrobial compound. Specifically, we investigated the activity of PMBN, a known Gram-negative outer membrane disrupting agent, via DCCAA. Lastly, through DCCAA we were able to monitor the intracellular accumulation of the d-cys conjugates of ciprofloxacin methyl ester, linezolid, puromycin, and rifamycin B.

We found all four antibiotic conjugates tested to display a signal above the background, indicating accumulation in the cytosol of bacteria. Through kinetic analysis, we found that the puromycin conjugate displayed a higher transport rate than the linezolid and ciprofloxacin-methyl-ester conjugates, while the rifamycin B conjugate displayed a distinct kinetic profile, unlike the other compounds, likely owing to a distinct membrane transport process or its larger size. We note that DCCAA does require the conjugation of a d-cysteine to the test molecule to make it compatible with luciferin generation. There must be some consideration of the location of this tag within the molecule (antibiotic) of interest. One option is to modify an existing amino group to then have the terminal amine of d-cysteine effectively replace that property within the molecules. Similar to the use of the fluorophore tag, one also has to consider the site within the molecule in consideration of the biological activity of the agent. Naturally, the physiochemical properties of the molecule could change with the addition of the tag, and these considerations are made in light of the ability to determine cytosolic arrival.

In conclusion, we propose that DCCAA can serve not only to elucidate the real-time intracellular accumulation dynamics of compounds tagged with a d-cys molecule in live bacterial cells but also to potentially unveil the membrane disruption abilities of untagged compounds. Furthermore, DCCAA can also be employed to reveal the presence and substrate specificity of esterase activity in live bacterial cells.

## Methods

### Transformation of FLUC2 pET28a into *E. coli*

For expression of the luciferase protein in *E. coli*, the FLUC2 pET28a plasmid was first transformed into DH5α *E. coli* cells for amplification. In short, 50 μL of competent DH5α *E. coli* cells and 2–5 μL of plasmid were added to an Eppendorf tube and kept on ice. After 30 min, a water bath was heated to 42 °C and the tube containing the cells and plasmid was placed in the bath for 30 s followed by another 2 min on ice. One mL of sterile LB medium was added to the tube, mixed, and transferred to a culture tube which was incubated at 37 °C for 1 h. Subsequently, 25–200 μL of cells from this tube were grown on LB/agar plates with kanamycin (50 μg/mL) at 37 °C overnight. Individual colonies were then picked from these plates and grown overnight in LB broth with 50 μg/mL kanamycin at 37 °C. The following day, a ZymoPURE Plasmid Miniprep Kit was used to extract the plasmid from the DH5α *E. coli* cells. A transformation was then performed as described above with competent BL21(DE3) *E. coli* cells for optimal expression. Glycerol stocks of both BL21 and DH5α *E. coli* cells were prepared by mixing 1 mL of overnight growth with 1 mL of 60% glycerol in water.

### Luciferase Protein Expression in *E. coli*

Sterile culture tubes each containing 3 mL of LB medium and 50 μg/mL kanamycin were inoculated with a stab of the transformed BL21 luciferase-expressing *E. coli* glycerol stock and incubated at 37 °C overnight. The next morning, the cells were diluted at a 1:10 ratio into fresh LB broth containing 50 μg/mL kanamycin and were grown at 37 °C for 3 h, or until the optical density at 600 nm reached a value between 0.6 and 0.8. Cultures were then induced with 1 mM isopropyl β-d-1-thiogalactopyranoside (IPTG) at 37 °C for 2 h in a shaker incubator to induce protein expression.

### Evaluation of Luciferase Protein Expression via SDS-PAGE

Overnight cultures of luciferase-expressing *E. coli* were grown and diluted the next morning as described above. When the diluted cells reached an OD600 between 0.6 and 0.8, IPTG was added to one culture tube to induce protein expression. Both plus and minus IPTG cultures were then incubated at 37 °C for 2 or 26 h. On the same day that these cells were induced, another two overnight cultures were grown. The next day, these cells were diluted, and IPTG was used to induce protein expression of one culture for 2 h at 37 °C. One mL of media was collected from the plus and minus IPTG samples from both time points. These were centrifuged at 3300*g* for 3 min in a HERAEUS multicentrifuge ×1 centrifuge (Thermo Fisher Scientific). The supernatant was discarded, and the cells were resuspended in 400 μL 1× phosphate buffered saline (PBS) at pH 7.2.40 μL of the cell resuspension was mixed with 10 μL of a 5X SDS-PAGE sample loading buffer. This mixture was then boiled for 5 min to denature the proteins. To run the gel, 8 μL of Thermo Scientific PageRuler Plus Prestained Protein Ladder, 10 to 250 kDa, was added to the first well. The samples were loaded at 20 μL, and then gel was run at 240 V for 30 min.

### Bioluminescence-Based Permeability Assays in Luciferase Expressing *E. coli*

Overnight cultures of luciferase-expressing *E. coli* were grown, and luciferase protein expression was induced as above. Subsequently, washing was performed by first pelleting the cells in the HERAEUS multicentrifuge ×1 centrifuge (Thermo Fisher Scientific) at 3300*g* for 3 min. The supernatant was then discarded, and the cells were resuspended in 1× PBS (volume same as original culture). This was repeated 2 times. The cells were resuspended after the last wash in 1× PBS. In NEM/PMBN pretreatment experiments, a 30 min incubation with the appropriate compound was performed at this point at the desired concentrations (100 μM for NEM; 0.5, 1, and 3 μM for PMBN). Cells were then washed and resuspended in 1× PBS in the same manner as described above. In a 96-well, black, flat-bottomed plate, the molecules of interest were then added to obtain the final desired concentration in a total volume of 100 μL. Note that stock solutions of molecules of interest were prepared in 1× PBS. 1× PBS was also added to any blank and negative control wells, to ensure the same total volume and number of cells in all the wells. A stock solution of CBT (6-Amino-2-cyanobenzothiazole) was prepared in *N*,*N*-Dimethylformamide (DMF), and appropriate volumes were added to the culture tubes containing cells to obtain the desired concentrations. Then, the same volume of cells was pipetted into each well, mixed, and placed in the BioTek Synergy H1Microplate Reader. The instrument was set to run on the end point/kinetic luminescence setting. Luminescence was set to read every 5 min for the run, usually for 60 min (or 120 min, for the extended time-course analysis). For the assay to test the luminescence of the pellet and supernatant of centrifuged *E. coli* cells, the plate was read for 30 min following which a set of wells treated with CBT and d-cystine were moved to another plate and pelleted as described above. The supernatant was then carefully separated and readded to the plate. The pellet was resuspended and also readded to the plate. Nonpelleted cells treated with CBT and d-cystine served as one of the controls. Luminescence in this case was then read for another 60 min. For all luminescence readings via the BioTek Synergy H1Microplate Reader, luminescence fiber was the optics type. The gain was set to 240, and the integration time was set to 0:01:00. The temperature was set to 37 °C.

### Cell-free Luciferase Assays

The 14.9 mg/mL luciferase enzyme stock received from Promega was diluted in 25 mM Tris buffer (pH 8) to prepare 0.4 mg/mL aliquots (40 μL each), which were then stored in −80 °C. All assays were performed in 25 mM Tris buffer (pH 8) in 96-well, black, flat-bottomed plates. Molecules of interest were added to obtain the final desired concentration in a total volume of 100 μL. Additionally, appropriate amounts of MgSO_4_ (100 mM), freshly made ATP (20 mM) and Luciferase stocks (0.4 mg/mL) prepared in 25 mM Tris buffer (pH 8) were added to the wells to yield final concentrations of 5 mM, 1 mM and 20 μg/mL, respectively. Wherever appropriate, a TCEP (20 mM) stock prepared in 25 mM Tris buffer (pH 8) was added to a final concentration of 1 mM. Similarly, wherever appropriate, a Coenzyme A (10 mM) stock prepared in 25 mM Tris buffer (pH 8) was added to a final concentration of 0.5 mM. In the experiments testing the effect of Coenzyme A, a porcine liver esterase stock [10 mg/mL in 25 mM Tris buffer (pH 8)] was added to yield a final concentration of 0.5 mg/mL. The plates were read using the BioTek Synergy H1Microplate Reader. The instrument was set to run on the end point/kinetic luminescence setting. Luminescence was set to read every 5 min for the run, usually between 30 min to 2 h. Luminescence fiber was the optics type. The gain was set to 240, and the integration time was set to 0:01:00. The temperature was set to 37 °C.

### CFU Analysis

Overnight cultures of luciferase-expressing *E. coli* were grown, and luciferase protein expression was induced as described above. Subsequently, washing was performed by first pelleting the cells in the HERAEUS Multicentrifuge ×1 centrifuge (Thermo Fisher Scientific) at 3300*g* for 2–3 min. The supernatant was then discarded, and the cells were resuspended in 1× PBS (volume same as original culture). This was repeated 2 times. The cells were resuspended after the last wash in 1× PBS. Resuspended cells were then incubated with the test compounds (CBT or PMBN) at the indicated concentrations for 30 min (PMBN) or 1 h (CBT). Cells were then washed and resuspended as described above. Serial dilutions of the cells were then carried out in PBS up to a dilution factor of 10^–8^. 80 μL of chosen dilutions was then plated on LB agar plates with 50 μg/mL kanamycin and incubated at 37 °C for 16 h. Subsequently, the number of colonies was manually counted.

### Nitrocefin Assay

Overnight cultures of luciferase-expressing *E. coli* were grown, and luciferase protein expression was induced as above to mimic conditions of the cells used in the bioluminescence assay. Cells were then washed twice and resuspended in 1× PBS and then incubated with the different concentrations of PMBN (same as those concentrations used in the bioluminescence assays) with 50 μg/mL nitrocefin for 30 min. As a negative control, cells were treated with PBS, and as a positive control, cells were treated with 10 mM EDTA for 30 min. At the end of the incubation period, the absorbance of the cells was read at 486 nm using the BioTek Synergy H1Microplate Reader. Untreated cells with no nitrocefin were treated as the blank. Wherever presented, the absorbances were blank-subtracted.

### SYTOX Green Assay

Overnight cultures of luciferase-expressing *E. coli* were grown, and luciferase protein expression was induced as above to mimic conditions of the cells used in the bioluminescence assay. Cells were then washed twice and resuspended in 1× PBS and then incubated with the test compounds (same concentrations as those used in the bioluminescence assays) for the appropriate time durations (30 min for PMBN and 1 h for antibiotic conjugates). Cells were then washed twice again and resuspended in 1× PBS. The experiment was then conducted as per the manufacturer’s protocol. As a negative control, cells were treated with PBS, and as a positive control, cells were treated with 10 mM EDTA. Cells were analyzed using an Attune NxT flow cytometer equipped with a 488 nm laser and 525/40 nm bandpass filter. The data were analyzed using the Attune NxT Software, where populations were gated and no less than 10,000 events per sample were recorded. Wherever presented, the mean fluorescence intensity (MFI) is the ratio of fluorescence levels above the negative control treatment.

## Supporting Information Available

Additional figures, tables, and materials/methods are included in the Supporting Information file (PDF)

## References

[ref1] Murray C. J. L.; Ikuta K. S.; Sharara F.; Swetschinski L.; Robles Aguilar G.; Gray A.; Han C.; Bisignano C.; Rao P.; Wool E.; Johnson S. C.; Browne A. J.; Chipeta M. G.; Fell F.; Hackett S.; Haines-Woodhouse G.; Kashef Hamadani B. H.; Kumaran E. A. P.; McManigal B.; Achalapong S.; Agarwal R.; Akech S.; Albertson S.; Amuasi J.; Andrews J.; Aravkin A.; Ashley E.; Babin F.-X.; Bailey F.; Baker S.; Basnyat B.; Bekker A.; Bender R.; Berkley J. A.; Bethou A.; Bielicki J.; Boonkasidecha S.; Bukosia J.; Carvalheiro C.; Castañeda-Orjuela C.; Chansamouth V.; Chaurasia S.; Chiurchiù S.; Chowdhury F.; Clotaire Donatien R.; Cook A. J.; Cooper B.; Cressey T. R.; Criollo-Mora E.; Cunningham M.; Darboe S.; Day N. P. J.; De Luca M.; Dokova K.; Dramowski A.; Dunachie S. J.; Duong Bich T.; Eckmanns T.; Eibach D.; Emami A.; Feasey N.; Fisher-Pearson N.; Forrest K.; Garcia C.; Garrett D.; Gastmeier P.; Giref A. Z.; Greer R. C.; Gupta V.; Haller S.; Haselbeck A.; Hay S. I.; Holm M.; Hopkins S.; Hsia Y.; Iregbu K. C.; Jacobs J.; Jarovsky D.; Javanmardi F.; Jenney A. W. J.; Khorana M.; Khusuwan S.; Kissoon N.; Kobeissi E.; Kostyanev T.; Krapp F.; Krumkamp R.; Kumar A.; Kyu H. H.; Lim C.; Lim K.; Limmathurotsakul D.; Loftus M. J.; Lunn M.; Ma J.; Manoharan A.; Marks F.; May J.; Mayxay M.; Mturi N.; Munera-Huertas T.; Musicha P.; Musila L. A.; Mussi-Pinhata M. M.; Naidu R. N.; Nakamura T.; Nanavati R.; Nangia S.; Newton P.; Ngoun C.; Novotney A.; Nwakanma D.; Obiero C. W.; Ochoa T. J.; Olivas-Martinez A.; Olliaro P.; Ooko E.; Ortiz-Brizuela E.; Ounchanum P.; Pak G. D.; Paredes J. L.; Peleg A. Y.; Perrone C.; Phe T.; Phommasone K.; Plakkal N.; Ponce-de-Leon A.; Raad M.; Ramdin T.; Rattanavong S.; Riddell A.; Roberts T.; Robotham J. V.; Roca A.; Rosenthal V. D.; Rudd K. E.; Russell N.; Sader H. S.; Saengchan W.; Schnall J.; Scott J. A. G.; Seekaew S.; Sharland M.; Shivamallappa M.; Sifuentes-Osornio J.; Simpson A. J.; Steenkeste N.; Stewardson A. J.; Stoeva T.; Tasak N.; Thaiprakong A.; Thwaites G.; Tigoi C.; Turner C.; Turner P.; van Doorn H. R.; Velaphi S.; Vongpradith A.; Vongsouvath M.; Vu H.; Walsh T.; Walson J. L.; Waner S.; Wangrangsimakul T.; Wannapinij P.; Wozniak T.; Young Sharma T. E. M. W.; Yu K. C.; Zheng P.; Sartorius B.; Lopez A. D.; Stergachis A.; Moore C.; Dolecek C.; Naghavi M. Global Burden of Bacterial Antimicrobial Resistance in 2019: A Systematic Analysis. Lancet 2022, 399 (10325), 629–655. 10.1016/S0140-6736(21)02724-0. 35065702 PMC8841637

[ref2] Hutchings M. I.; Truman A. W.; Wilkinson B. Antibiotics: Past, Present and Future. Curr. Opin. Microbiol. 2019, 51, 72–80. 10.1016/j.mib.2019.10.008. 31733401

[ref3] Zgurskaya H. I.; Rybenkov V. V. Permeability Barriers of Gram-Negative Pathogens. Ann. N.Y. Acad. Sci. 2020, 1459 (1), 5–18. 10.1111/nyas.14134. 31165502 PMC6940542

[ref4] Kohanski M. A.; Dwyer D. J.; Collins J. J. How Antibiotics Kill Bacteria: From Targets to Networks. Nat. Rev. Microbiol. 2010, 8 (6), 423–435. 10.1038/nrmicro2333. 20440275 PMC2896384

[ref5] Kojima S.; Nikaido H. Permeation Rates of Penicillins Indicate That Escherichia Coli Porins Function Principally as Nonspecific Channels. Proc. Natl. Acad. Sci. U.S.A. 2013, 110 (28), E2629–E2634. 10.1073/pnas.1310333110. 23798411 PMC3710850

[ref6] June C. M.; Vaughan R. M.; Ulberg L. S.; Bonomo R. A.; Witucki L. A.; Leonard D. A. A fluorescent carbapenem for structure function studies of penicillin-binding proteins, β-lactamases, and β-lactam sensors. Anal. Biochem. 2014, 463, 70–74. 10.1016/j.ab.2014.07.012. 25058926 PMC4167909

[ref7] Davis T. D.; Gerry C. J.; Tan D. S. General Platform for Systematic Quantitative Evaluation of Small-Molecule Permeability in Bacteria. ACS Chem. Biol. 2014, 9 (11), 2535–2544. 10.1021/cb5003015. 25198656 PMC4245172

[ref8] Ghai I.; Winterhalter M.; Wagner R. Probing transport of charged β-lactamase inhibitors through OmpC, a membrane channel from E. coli. Biochem. Biophys. Res. Commun. 2017, 484 (1), 51–55. 10.1016/j.bbrc.2017.01.076. 28109883

[ref9] Kaščáková S.; Maigre L.; Chevalier J.; Réfrégiers M.; Pagès J. M. Antibiotic Transport in Resistant Bacteria: Synchrotron UV Fluorescence Microscopy to Determine Antibiotic Accumulation with Single Cell Resolution. PLoS One 2012, 7 (6), e38624 10.1371/journal.pone.0038624. 22719907 PMC3373604

[ref10] Zhou Y.; Joubran C.; Miller-Vedam L.; Isabella V.; Nayar A.; Tentarelli S.; Miller A. Thinking Outside the “Bug”: A Unique Assay To Measure Intracellular Drug Penetration in Gram-Negative Bacteria. Anal. Chem. 2015, 87 (7), 3579–3584. 10.1021/ac504880r. 25753586

[ref11] Cama J.; Bajaj H.; Pagliara S.; Maier T.; Braun Y.; Winterhalter M.; Keyser U. F. Quantification of Fluoroquinolone Uptake through the Outer Membrane Channel OmpF of Escherichia Coli. J. Am. Chem. Soc. 2015, 137 (43), 13836–13843. 10.1021/jacs.5b08960. 26478537

[ref12] Cinquin B.; Maigre L.; Pinet E.; Chevalier J.; Stavenger R. A.; Mills S.; Réfrégiers M.; Pagès J. M. Microspectrometric Insights on the Uptake of Antibiotics at the Single Bacterial Cell Level. Sci. Rep. 2015, 5 (1), 17968 10.1038/srep17968. 26656111 PMC4675965

[ref13] Geddes E. J.; Li Z.; Hergenrother P. J. An LC-MS/MS Assay and Complementary Web-Based Tool to Quantify and Predict Compound Accumulation in E. Coli. Nat. Protoc. 2021, 16 (10), 4833–4854. 10.1038/s41596-021-00598-y. 34480129 PMC8715754

[ref14] Iyer R.; Ye Z.; Ferrari A.; Duncan L.; Tanudra M. A.; Tsao H.; Wang T.; Gao H.; Brummel C. L.; Erwin A. L. Evaluating LC-MS/MS To Measure Accumulation of Compounds within Bacteria. ACS Infect. Dis. 2018, 4 (9), 1336–1345. 10.1021/acsinfecdis.8b00083. 29961312

[ref15] Jones S. W.; Christison R.; Bundell K.; Voyce C. J.; Brockbank S. M. V.; Newham P.; Lindsay M. A. Characterisation of Cell-Penetrating Peptide-Mediated Peptide Delivery. Br. J. Pharmacol. 2005, 145 (8), 1093–1102. 10.1038/sj.bjp.0706279. 15937518 PMC1576229

[ref16] Fischer R.; Waizenegger T.; Köhler K.; Brock R. A Quantitative Validation of Fluorophore-Labelled Cell-Permeable Peptide Conjugates: Fluorophore and Cargo Dependence of Import. Biochim. Biophys. Acta, Biomembr. 2002, 1564 (2), 365–374. 10.1016/S0005-2736(02)00471-6. 12175919

[ref17] Illien F.; Rodriguez N.; Amoura M.; Joliot A.; Pallerla M.; Cribier S.; Burlina F.; Sagan S. Quantitative Fluorescence Spectroscopy and Flow Cytometry Analyses of Cell-Penetrating Peptides Internalization Pathways: Optimization, Pitfalls, Comparison with Mass Spectrometry Quantification. Sci. Rep. 2016, 6 (1), 36938 10.1038/srep36938. 27841303 PMC5107916

[ref18] Liu Z.; Lepori I.; Chordia M. D.; Dalesandro B. E.; Guo T.; Dong J.; Siegrist M. S.; Pires M. M. A Metabolic-Tag-Based Method for Assessing the Permeation of Small Molecules Across the Mycomembrane in Live Mycobacteria**. Angew. Chem., Int. Ed. 2023, 62 (20), e202217777 10.1002/anie.202217777. PMC10159989 36700874

[ref19] Kelly J. J.; Dalesandro B. E.; Liu Z.; Chordia M. D.; Ongwae G. M.; Pires M. M. Measurement of Accumulation of Antibiotics to Staphylococcus Aureus in Phagosomes of Live Macrophages. Angew. Chem. 2024, 136 (3), e202313870 10.1002/ange.202313870. PMC10799677 38051128

[ref20] Ongwae G. M.; Lepori I.; Chordia M. D.; Dalesandro B. E.; Apostolos A. J.; Siegrist M. S.; Pires M. M. Measurement of Small Molecule Accumulation into Diderm Bacteria. ACS Infect. Dis. 2023, 9 (1), 97–110. 10.1021/acsinfecdis.2c00435. 36530146

[ref21] Karatas H.; Maric T.; D’Alessandro P. L.; Yevtodiyenko A.; Vorherr T.; Hollingworth G. J.; Goun E. A. Real-Time Imaging and Quantification of Peptide Uptake in Vitro and in Vivo. ACS Chem. Biol. 2019, 14 (10), 2197–2205. 10.1021/acschembio.9b00439. 31498986

[ref22] Godinat A.; Bazhin A. A.; Goun E. A. Bioorthogonal Chemistry in Bioluminescence Imaging. Drug Discovery Today 2018, 23 (9), 1584–1590. 10.1016/j.drudis.2018.05.022. 29778694

[ref23] Carmel-Harel O.; Storz G. Roles of the Glutathione- and Thioredoxin-Dependent Reduction Systems in the Escherichia Coli and Saccharomyces Cerevisiae Responses to Oxidative Stress. Annu. Rev. Microbiol. 2000, 54 (1), 439–461. 10.1146/annurev.micro.54.1.439. 11018134

[ref24] Patterson D. M.; Nazarova L. A.; Prescher J. A. Finding the Right (Bioorthogonal) Chemistry. ACS Chem. Biol. 2014, 9 (3), 592–605. 10.1021/cb400828a. 24437719

[ref25] Liang G.; Ren H.; Rao J. A Biocompatible Condensation Reaction for Controlled Assembly of Nanostructures in Living Cells. Nat. Chem. 2010, 2 (1), 54–60. 10.1038/nchem.480. 21124381 PMC3196337

[ref26] Nguyen D. P.; Elliott T.; Holt M.; Muir T. W.; Chin J. W. Genetically Encoded 1,2-Aminothiols Facilitate Rapid and Site-Specific Protein Labeling via a Bio-Orthogonal Cyanobenzothiazole Condensation. J. Am. Chem. Soc. 2011, 133 (30), 11418–11421. 10.1021/ja203111c. 21736333

[ref27] Ramil C. P.; An P.; Yu Z.; Lin Q. Sequence-Specific 2-Cyanobenzothiazole Ligation. J. Am. Chem. Soc. 2016, 138 (17), 5499–5502. 10.1021/jacs.6b00982. 27082895 PMC4861237

[ref28] Ren H.; Xiao F.; Zhan K.; Kim Y.-P.; Xie H.; Xia Z.; Rao J. A Biocompatible Condensation Reaction for the Labeling of Terminal Cysteine Residues on Proteins. Angew. Chem., Int. Ed. 2009, 48 (51), 9658–9662. 10.1002/anie.200903627. PMC4878437 19924746

[ref29] Van de Bittner G. C.; Bertozzi C. R.; Chang C. J. Strategy for Dual-Analyte Luciferin Imaging: In Vivo Bioluminescence Detection of Hydrogen Peroxide and Caspase Activity in a Murine Model of Acute Inflammation. J. Am. Chem. Soc. 2013, 135 (5), 1783–1795. 10.1021/ja309078t. 23347279 PMC3583381

[ref30] Roychaudhuri R.; Gadalla M. M.; West T.; Snyder S. H. A Novel Stereospecific Bioluminescent Assay for Detection of Endogenous D-Cysteine. ACS Chem. Neurosci. 2022, 13 (23), 3257–3262. 10.1021/acschemneuro.2c00528. 36403160

[ref31] Semenza E. R.; Harraz M. M.; Abramson E.; Malla A. P.; Vasavda C.; Gadalla M. M.; Kornberg M. D.; Snyder S. H.; Roychaudhuri R. D-Cysteine Is an Endogenous Regulator of Neural Progenitor Cell Dynamics in the Mammalian Brain. Proc. Natl. Acad. Sci. U.S.A. 2021, 118 (39), e2110610118 10.1073/pnas.2110610118. 34556581 PMC8488618

[ref32] Zhang B. S.; Jones K. A.; McCutcheon D. C.; Prescher J. A. Pyridone Luciferins and Mutant Luciferases for Bioluminescence Imaging. ChemBioChem 2018, 19 (5), 470–477. 10.1002/cbic.201700542. 29384255 PMC6163054

[ref33] Xiong Y.; Zhang Y.; Li Z.; Reza M. S.; Li X.; Tian X.; Ai H. Engineered Amber-Emitting Nano Luciferase and Its Use for Immunobioluminescence Imaging In Vivo. J. Am. Chem. Soc. 2022, 144 (31), 14101–14111. 10.1021/jacs.2c02320. 35913786 PMC9425369

[ref34] White E. H.; Wörther H.; Seliger H. H.; McElroy W. D. Amino Analogs of Firefly Luciferin and Biological Activity Thereof1. J. Am. Chem. Soc. 1966, 88 (9), 2015–2019. 10.1021/ja00961a030.

[ref35] Godinat A.; Budin G.; Morales A. R.; Park H. M.; Sanman L. E.; Bogyo M.; Yu A.; Stahl A.; Dubikovskaya E. A. A Biocompatible “Split Luciferin” Reaction and Its Application for Non-Invasive Bioluminescent Imaging of Protease Activity in Living Animals. Curr. Protoc. Chem. Biol. 2014, 6 (3), 169–189. 10.1002/9780470559277.ch140047. 25205565 PMC4219325

[ref36] Nakamura M.; Niwa K.; Maki S.; Hirano T.; Ohmiya Y.; Niwa H. Construction of a New Firefly Bioluminescence System Using L-Luciferin as Substrate. Tetrahedron Lett. 2006, 47 (7), 1197–1200. 10.1016/j.tetlet.2005.12.033.

[ref37] Niwa K.; Nakamura M.; Ohmiya Y. Stereoisomeric Bio-Inversion Key to Biosynthesis of Firefly d-Luciferin. FEBS Lett. 2006, 580 (22), 5283–5287. 10.1016/j.febslet.2006.08.073. 16979628

[ref38] Ren Y.; Qiang Y.; Zhu B.; Tang W.; Duan X.; Li Z. General Strategy for Bioluminescence Sensing of Peptidase Activity In Vivo Based on Tumor-Targeting Probiotic. Anal. Chem. 2021, 93 (9), 4334–4341. 10.1021/acs.analchem.1c00093. 33624497

[ref39] Niwa K.; Nakajima Y.; Ohmiya Y. Applications of Luciferin Biosynthesis: Bioluminescence Assays for l-Cysteine and Luciferase. Anal. Biochem. 2010, 396 (2), 316–318. 10.1016/j.ab.2009.09.014. 19748476

[ref40] Nakamura M.; Maki S.; Amano Y.; Ohkita Y.; Niwa K.; Hirano T.; Ohmiya Y.; Niwa H. Firefly Luciferase Exhibits Bimodal Action Depending on the Luciferin Chirality. Biochem. Biophys. Res. Commun. 2005, 331 (2), 471–475. 10.1016/j.bbrc.2005.03.202. 15850783

[ref41] Fahey R. C.; Brown W. C.; Adams W. B.; Worsham M. B. Occurrence of Glutathione in Bacteria. J. Bacteriol. 1978, 133 (3), 1126–1129. 10.1128/jb.133.3.1126-1129.1978. 417060 PMC222142

[ref42] Taylor M. D. Improved Passive Oral Drug Delivery via Prodrugs. Adv. Drug Delivery Rev. 1996, 19 (2), 131–148. 10.1016/0169-409X(95)00104-F.

[ref43] Beaumont K.; Webster R.; Gardner I.; Dack K. Design of Ester Prodrugs to Enhance Oral Absorption of Poorly Permeable Compounds: Challenges to the Discovery Scientist. Curr. Drug Metab. 2003, 4 (6), 461–485. 10.2174/1389200033489253. 14683475

[ref44] Mohammad I.; Liebmann K. L.; Miller S. C. Firefly Luciferin Methyl Ester Illuminates the Activity of Multiple Serine Hydrolases. Chem. Commun. 2023, 59 (55), 8552–8555. 10.1039/D3CC02540C. PMC10347678 37337906

[ref45] Antonczak A. K.; Simova Z.; Tippmann E. M. A Critical Examination of Escherichia Coli Esterase Activity *. J. Biol. Chem. 2009, 284 (42), 28795–28800. 10.1074/jbc.M109.027409. 19666472 PMC2781425

[ref46] Brown A. R.; Wodzanowski K. A.; Santiago C. C.; Hyland S. N.; Follmar J. L.; Asare-Okai P.; Grimes C. L. Protected N-Acetyl Muramic Acid Probes Improve Bacterial Peptidoglycan Incorporation via Metabolic Labeling. ACS Chem. Biol. 2021, 16 (10), 1908–1916. 10.1021/acschembio.1c00268. 34506714 PMC8722712

[ref47] Korshunov S.; Imlay K. R. C.; Imlay J. A. Cystine Import Is a Valuable but Risky Process Whose Hazards Escherichia Coli Minimizes by Inducing a Cysteine Exporter. Mol. Microbiol. 2020, 113 (1), 22–39. 10.1111/mmi.14403. 31612555 PMC7007315

[ref48] Chonoles Imlay K. R.; Korshunov S.; Imlay J. A. Physiological Roles and Adverse Effects of the Two Cystine Importers of Escherichia Coli. J. Bacteriol. 2015, 197 (23), 3629–3644. 10.1128/JB.00277-15. 26350134 PMC4626903

[ref49] French S.; Farha M.; Ellis M. J.; Sameer Z.; Côté J. P.; Cotroneo N.; Lister T.; Rubio A.; Brown E. D. Potentiation of Antibiotics against Gram-Negative Bacteria by Polymyxin B Analogue SPR741 from Unique Perturbation of the Outer Membrane. ACS Infect. Dis. 2020, 6 (6), 1405–1412. 10.1021/acsinfecdis.9b00159. 31566948

[ref50] Ofek I.; Cohen S.; Rahmani R.; Kabha K.; Tamarkin D.; Herzig Y.; Rubinstein E. Antibacterial Synergism of Polymyxin B Nonapeptide and Hydrophobic Antibiotics in Experimental Gram-Negative Infections in Mice. Antimicrob. Agents Chemother. 1994, 38 (2), 374–377. 10.1128/AAC.38.2.374. 8192470 PMC284461

[ref51] Dixon R. A.; Chopra I. Polymyxin B and Polymyxin B Nonapeptide Alter Cytoplasmic Membrane Permeability in Escherichia Coli. J. Antimicrob. Chemother. 1986, 18 (5), 557–563. 10.1093/jac/18.5.557. 3027012

[ref52] Lomakina G. Yu.; Ugarova N. N. Kinetics of the Interaction of Colistin with Live Escherichia Coli Cells by the Bioluminescence Method. Mosc. Univ. Chem. Bull. 2022, 77 (1), 42–47. 10.3103/S0027131422010059.

[ref53] Ihssen J.; Jovanovic N.; Sirec T.; Spitz U. Real-Time Monitoring of Extracellular ATP in Bacterial Cultures Using Thermostable Luciferase. PLoS One 2021, 16 (1), e0244200 10.1371/journal.pone.0244200. 33481792 PMC7822345

[ref54] Branchini B. R.; Magyar R. A.; Murtiashaw M. H.; Anderson S. M.; Zimmer M. Site-Directed Mutagenesis of Histidine 245 in Firefly Luciferase: A Proposed Model of the Active Site. Biochemistry 1998, 37 (44), 15311–15319. 10.1021/bi981150d. 9799491

[ref55] Wilhelm M. J.; Sharifian Gh M.; Dai H.-L. Chemically Induced Changes to Membrane Permeability in Living Cells Probed with Nonlinear Light Scattering. Biochemistry 2015, 54 (29), 4427–4430. 10.1021/acs.biochem.5b00600. 26122620

[ref56] Wilhelm M. J.; Sharifian Gh M.; Dai H.-L. Influence of Molecular Structure on Passive Membrane Transport: A Case Study by Second Harmonic Light Scattering. J. Chem. Phys. 2019, 150 (10), 104705 10.1063/1.5081720. 30876365

